# Supplementary material for: Cyclodextrin Complexation of Fenofibrate by Co-Grinding Method and Monitoring the Process Using Complementary Analytical Tools
Source: Pharmaceutics. 2022 Jun 23;14(7):1329. doi: 10.3390/pharmaceutics14071329 (PMC9319411; doi:10.3390/pharmaceutics14071329)
Supplement: Supplementary file 1 [file pharmaceutics-14-01329-s001.zip › pharmaceutics-1747617-supplementary.pdf]

Figure S1 shows thermograms of the API and the excipient obtained by DSC measurements. FEN Form-I polymorph has a characteristic endothermic peak at *ca* 80.5 °C. An endothermic signal (between 25-85 °C) is observed for the CD, indicating the presence of water.

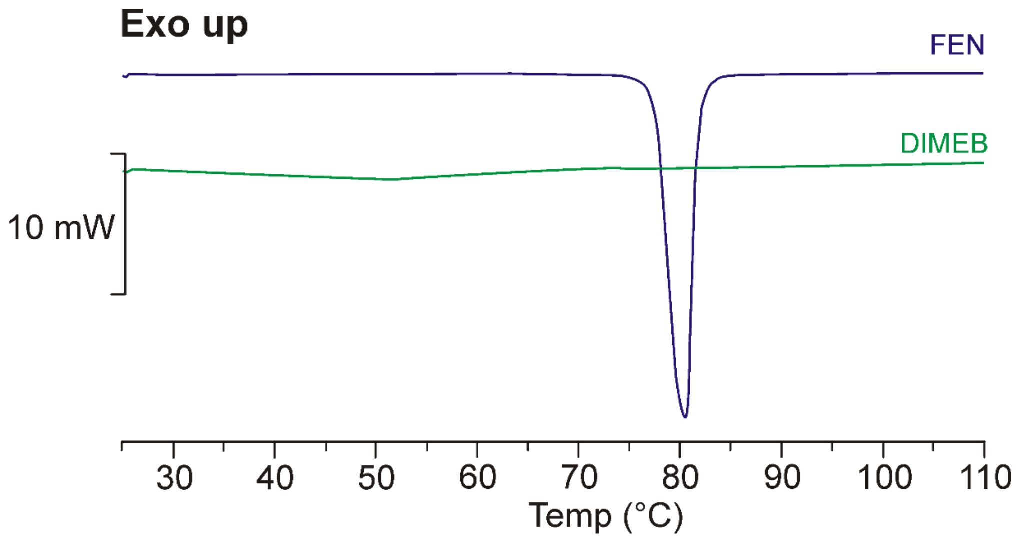

Figure S1. DSC thermograms of FEN and DIMEB.

XRPD measurements were performed with the parameters several times. Figure S2 shows the diffractograms of products immediately after the products are prepared and after 2 years. The products retained their amorphous properties during this long stability test.

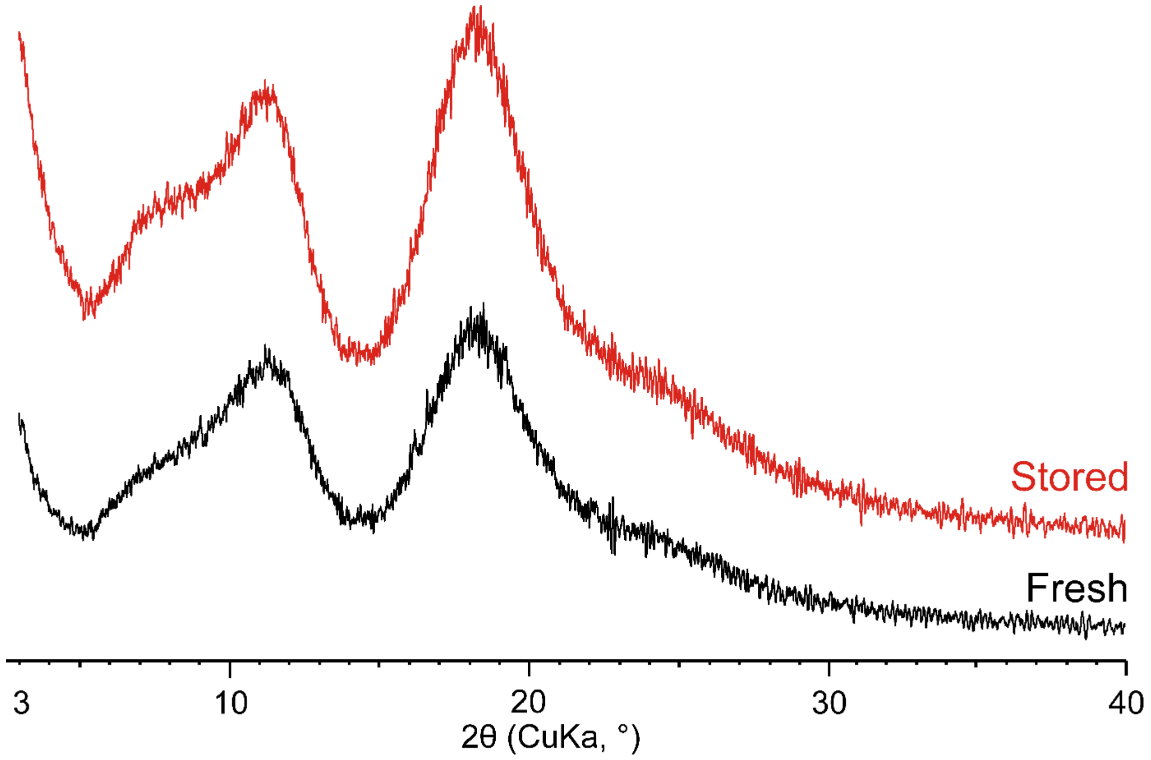

Figure S2. XRPD diffractograms of products after production and after 2 years of storage.

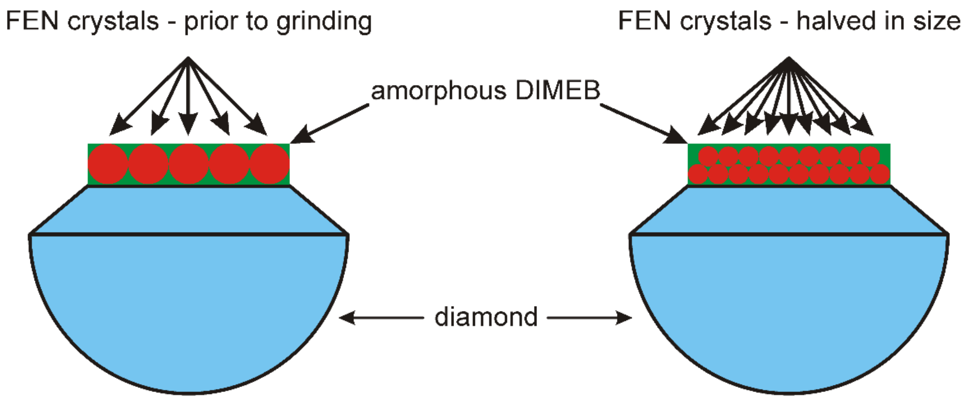

Figure S3. Schematic explanation of the changes in the relative intensities of FEN (red) and DIMEB (green) in the ATR spectra measured during the first 20 minutes of grinding. The same amount of material, distributed in particles smaller in size, increases the contact surface with the ATR element causing increased relative intensity in the recorded spectrum.

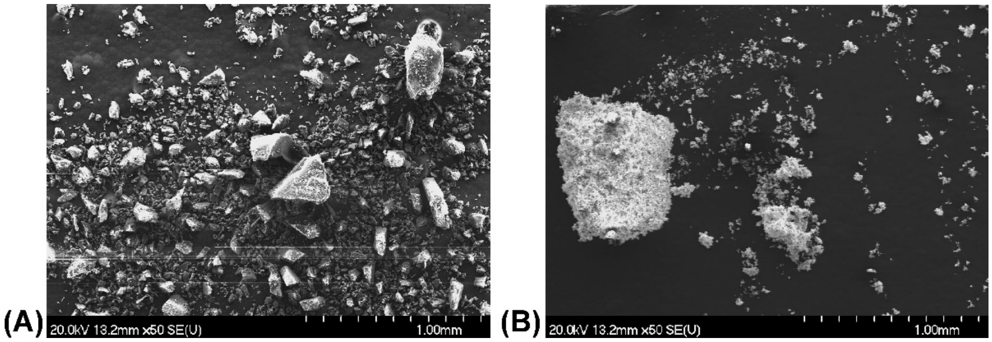

Figure S4. SEM images of FEN (A) and DIMEB (B), at magnification of 50, clearly showed the differences in crystallinity and the size of particles of the starting materials.

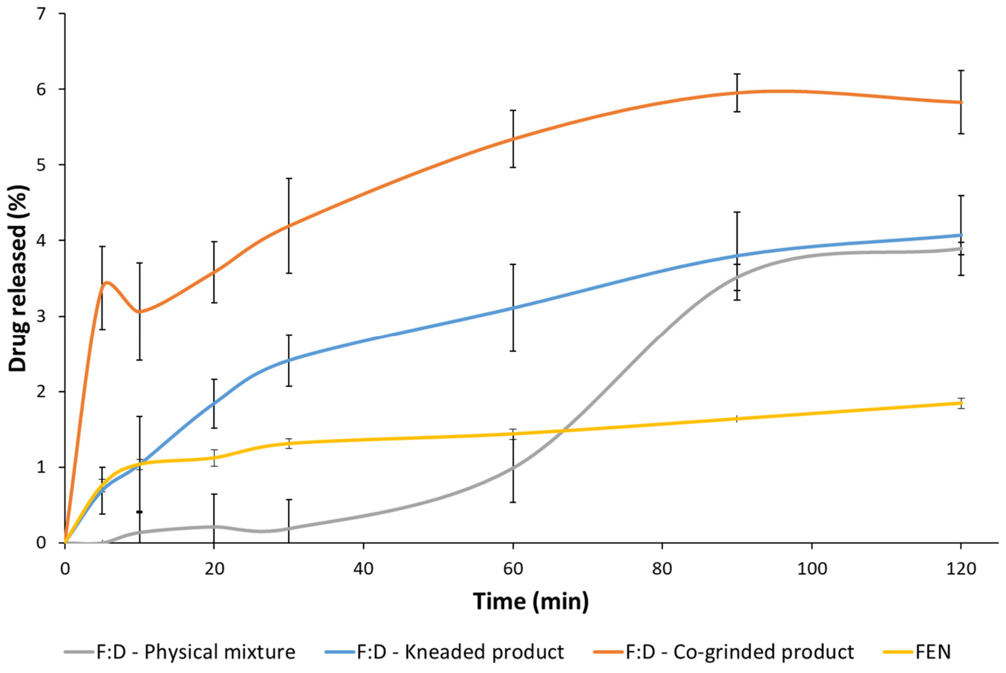

Figure S5. Diffusion curves of FEN, FEN:DIMEB (F:D) physical mixture, kneaded product, and co-ground product. Best diffusion properties were achieved by ground product.

Table S1. Assignment of peaks of FEN based on the literature data.

Peak position/ cm^{-1}	Description	Assignment* [6]	Assignment [5]	Assignment	References
1729	s	$\nu_{C=O}$ – R-C=O OR’	$\nu_{C=O}$ – R-C=O OR’	C=O stretching of ester group	[1] [3] [4]
1651	s	$\nu_{C=O}$ – $(Ar)_2$>C=O	$\nu_{C=O}$ – Ar-C=O	C=O stretching of carbonyl group	[1] [3] [4]
1599	s	*8a* -O-	aromatic ring stretching	8a and 8b mode of 1,4-O and C=O subst. ring	[1]
1588	m	*8a* Cl-	aromatic ring stretching	8a and 8b mode of 1,4-Cl and C=O subst. ring	[1]
1572	w	*8b* X-	-	in-plane ring def. mode of 1,4-O and C=O subst. ring- amorfous	[1]
1563	w	*8b*	-	in-plane ring def. mode of 1,4-O	[1]

		X-		and C=O subst. ring - crystalline	
1503	m	*19a* -O-	aromatic ring stretching + C-H bending	ring stretch.	[3]
1486	w	*19a* Cl-	-		
1467	w	$\delta_{as}^{+} CH_3$	>$C(CH_3)_2$ asym. out of phase bending		
1451	w				
1439	vw				
1419	m	*19b* -O-	aromatic ring stretching + C-H bending	in-plane ring def. mode of 1,4-O and C=O subst. ring	[1]
1398	w	*19b* Cl-	-	-CH3 in-phase bending	[5]
1385	m	$\delta_s\ CH_3$	-	-CH3 in-phase bending	[5]
1377	w	$\delta_s\ CH_3$	>$C(CH_3)_2$ symmetric in phase bendings		
1368	w	$\delta_s\ CH_3$			
1345	vw	$\delta_s\ CH_3$			
1302	s	*14* Cl-	Ar-O-C stretching		
1287	s	*3* Cl-		aryl-ether	[3]
1276	m	*3* -O-		asym. C-C-O stretch. - ester group	[1]
1249	s	-		asym. C-C-O stretch. - ring-O- /asym. C-C(=O)-C stretch. |ring in-plane C-H def.	[1] | [3]
1205	vw	-			
1183	s	*9a* -O-	-	in-plane C-H bending of O=C-ring-O-	[1]
1174	s	-	-	ring in-plane C-H def.	[3]
1159	s	-	-CH_3 in plane rocking		
1145	s	-		C-O-ring stretch.	[1] [3]
1117	m	*18b* -O-	in plane aromatic ring deformations	ring in-plane C-H def.	[3]
1101	s	*18b* Cl-		$(CH_3)_2$-HC-O- stretch.	[1]
1088	s	*1* Cl-		Cl-ring stretch.	[1] [4]
1013	m	*18a* Cl-		ring in-plane C-H def.	[3]
975	m	ν_{C-C}	-	ring in-plane C-H def.	[3]
925	s	-	-CH_3 in plane rocking		
899	vw	-			

860	m	*17b*<br>X-		ring out of plane C-H def.	[3]
844	m	*17b*<br>X-	out of plane aromatic ring deformations	ring out of plane C-H def.	[3]
824	w	*1†*<br>O-			
819	w	*1†*<br>O-		O-CH<(CH3)2	[2]
765	s	*6a*<br>Cl-	-		
740	w	-			
718	vw	*4*<br>Cl-	out of plane aromatic ring deformations		
683	w	*4†*<br>-O-		ring out of plane C-H def.	[3]
656	m	*4†*<br>-O-			
637	vw	-	-		
626	vw	-	-		

** Based on the assignment of p-methoxy acetopenone and p-chloroacetophenon, using Wilson's notation for aromatic ring vibrations. The para substituent Cl- or -O- was given, when it was possible, and X- when it was uncertain.*

† Either was possible to assign.

References:

[1] Heinz, A.; Gordon, K.C.; McGoverin, C.M.; Rades, T.; Strachan, C.J. Understanding the Solid-State Forms of Fenofibrate - A Spectroscopic and Computational Study. *Eur. J. Pharm. Biopharm.* 2009, *71*, 100–108.

[2] P.J.Larkin. *IR and Raman Spectroscopy, Principles and Spectral Interpretation* Elsevier Inc., Oxford 2011

[3] Tipduangta, P.; Takieddin, K.; Fabian, L.; Belton, P.; Qi, S. Towards Controlling the Crystallisation Behaviour of Fenofibrate Melt: Triggers of Crystallisation and Polymorphic Transformation. *Rsc Advances* **2018**, 8, 13513–13525.

[4] Vazquez, I.N.; Rodriguez-Nunez, J.R.; Pena-Caballero, V.; Ruvalcaba, R.M.; Aceves-Hernandez, J.M. Theoretical and Experimental Study of Fenofibrate and Simvastatin. *J. Mol. Struct.* 2017, *1149*, 683–693.

[5] Colthup, N.B.; Daly, L.H.; Wieberley, S.E. *Introduction to Infrared and Raman Spectroscopy.* Academic Press: New York, 1990. pp. 218,236,250, 261-283.

[6] Varsányi, György. *Assignments for vibrational spectra of seven hundred benzene derivatives*; Akadémiai Kiadó: Budapest, 1973; pp. 12, 128, 163, 550–551.
